# mSphere of Influence: Targeting bacterial signaling and metabolism to overcome antimicrobial resistance

**DOI:** 10.1128/msphere.00632-23

**Published:** 2024-02-02

**Authors:** Merve S. Zeden

**Affiliations:** 1 School of Biological and Chemical Sciences, College of Science and Engineering, University of Galway, Galway, Ireland; University of Michigan, Ann Arbor, Michigan, USA

**Keywords:** bacterial signalling, *Staphylococcus aureus*, *Listeria monocytogenes*, AMR, MRSA, ESKAPE, central metabolism, antibiotic resistance, beta-lactams, c-di-AMP

## Abstract

Dr Merve Suzan Zeden works in the field of molecular bacteriology and antibiotic resistance. In this mSphere of Influence article, she reflects on how three papers, entitled “c-di-AMP modulates *Listeria monocytogenes* central metabolism to regulate growth, antibiotic resistance and osmoregulation,” “Amino acid catabolism in *Staphylococcus aureus* and the function of carbon catabolite repression,” and “Evolving MRSA: high-level β-lactam resistance in *Staphylococcus aureus* is associated with RNA polymerase alterations and fine tuning of gene expression,” made an impact on her work on bacterial metabolism and antimicrobial resistance and how it shaped her research in understanding the link in between.

## COMMENTARY

Bacteria have a remarkable ability to adapt to multiple niches and thrive under diverse environmental stimuli by fine-tuning their biological processes. Antibiotics have long represented a successful form of therapy in medicine. However, experts predict that antimicrobial resistance (AMR) due to infections by so-called “superbugs” could kill 10 million people annually by 2050 (1). Therefore, research on understanding how bacteria prevail in their respective niches fascinates me in the hope that we will find alternative solutions in the fight against AMR. In this commentary, I emphasize work from three research groups that overlap in their aspiration to understand bacterial metabolism and/or antimicrobial resistance. These are “c-di-AMP modulates *Listeria monocytogenes* central metabolism to regulate growth, antibiotic resistance and osmoregulation” by Whiteley et al. (2), “Amino acid catabolism in *Staphylococcus aureus* and the function of carbon catabolite repression” by Halsey et al. (3), and “Evolving MRSA: high-level β-lactam resistance in *Staphylococcus aureus* is associated with RNA polymerase alterations and fine tuning of gene expression” by Panchal et al. (4). These three papers have influenced my view on the way we study bacterial metabolism, including the mechanisms by which they persist in particular niches and how this pertains to AMR.

The second messenger c-di-AMP is predominantly found in gram-positive bacteria and is fundamental for several processes in bacteria, including their ability to cope with harsh conditions like high-saline environments, and modulating cell wall synthesis in response to cell envelope damage, acid stress, and heat stress (5). Elegant genetic work by Whiteley et al. elucidated the role of c-di-AMP in both beta-lactam resistance and central metabolism in *L. monocytogenes* (2, 6). Comparison of growth by wild-type (WT) *L. monocytogenes* and a Δ*dacA* mutant lacking the diadenylate cyclase (c-di-AMP synthase) enzyme revealed a significant growth defect in brain heart infusion medium but not in chemically defined Listeria synthetic medium (2). The Δ*dacA* mutant also exhibited dramatically increased sensitivity to cefuroxime, which was reversed by suppressor mutations in oligopeptide transporters as well as central metabolic genes (2). This paper clearly revealed that the regulation of bacterial osmoregulation and metabolism by c-di-AMP, which is profoundly influenced by growth conditions, plays an important role in resistance to beta-lactam antibiotics (2). Extrapolating from the impact of rich versus minimal media on these phenotypes prompted me to investigate how significant alterations in bacterial metabolism impact growth and antimicrobial drug resistance. In *S. aureus*, c-di-AMP has been shown to play a role in potassium, amino acid, and osmolyte transport (7–9). In *L. monocytogenes,* accumulation of citrate in a Δ*dacA* mutant indicated significant changes at the pyruvate node of central metabolism (2). The intricate regulatory mechanisms involving signaling nucleotides, and the metabolic adaptability that underpins resistance phenotypes cannot be ignored in our global efforts in the fight against AMR.

Research by Halsey et al. added further to my increasing awareness about metabolism and how *S. aureus* possesses a spectacular ability for environmental adaptation (3). Halsey et al. demonstrated beautifully how *S. aureus* can redirect its metabolism to cope with the absence of its favorite carbon source, glucose, by relying on amino acid uptake (3). Within a staphylococcal abscess, glucose can become limiting; thus, *S. aureus* utilizes its ability to catabolize secondary carbon sources, like amino acids, to survive. Using the sequence-defined Nebraska transposon library (10) facilitated the analysis of strains with mutations in amino acid catabolism genes, and nuclear magnetic resonance highlighted the requirement for specific amino acids for growth including in late exponential and stationary phase (3). Alanine, serine, glycine, threonine, arginine, proline, glutamate, and aspartate were preferentially consumed from chemically defined media (CDM) within first 8 hours (3). Further analysis revealed that some mutations in metabolic genes did not have the ability to thrive or grow at all in CDM (3), which was intriguing, given that Δ*dacA* mutants actually prefer CDM over rich media (11). That said, it is important to note that Δ*dacA* mutants are not directly comparable to the metabolic mutants studied by Halsey et al. Overall, this work gave me a great appreciation for how *S. aureus* adaptation to various niches is underpinned by metabolic changes that are fluidic and constantly fluctuating. This study also influenced me in the discovery of purine nucleosides as potential beta-lactam adjuvants in *S. aureus* (12). By using CDM recipe from Halsey et al., with and without purine supplementation, we elucidated the role of purine homeostasis in beta-lactam resistance (12). Uncovering the metabolic adaptability of *S. aureus* in niche-specific models of infection under controlled growth conditions and understanding how and when amino acids or other nutrients are transported into *S. aureus* cells may help us identify specific druggable targets for the development of novel therapeutics against staphylococcal infections (13, 14).

Research into antibiotic resistance mechanisms frequently highlights how the lucrative ways that bacteria can become resistant are the result of changes in metabolism or signaling (15–17). The 2020 Panchal et al. paper helped me appreciate the immense adaptability of bacteria in response to antibiotic stress. They introduced the essential methicillin resistance gene *mecA* onto the chromosome of the susceptible *S. aureus* strain SH1000 and “trained” it to become a highly resistant MRSA, thereby revealing new insights into resistance. Training this strain to express increased resistance was achieved by plating onto increasing concentrations of oxacillin. Mutations associated with high-level resistance were identified in the RNA polymerase genes *rpoB* and *rpoC,* which appear to genetically fine-tune the metabolic and physiological changes required for this phenotype. This work has significantly enhanced our understanding of how bacteria can evolve in the presence of antibiotics. Moreover, this research shows how the presence of antibiotics in our environment may open up opportunities for tolerance and resistance mechanisms to emerge. Intriguingly, mutations in *rpoB* have been shown to lead to a stringent-like response that also increases resistance to rifampicin and ciprofloxacin (18, 19). The stringent response alarmone (p)ppGpp is required for high-level beta-lactam resistance and represses the activity of the c-di-AMP phosphodiesterase GdpP (6, 15, 18–23).

The principles that have emerged from these three papers, as well as many others in signaling, metabolism, and resistance fields (6, 7, 16, 20, 22–46), have greatly inspired and influenced the way I do my research and the way I think about how metabolism and resistance are interlinked. My publications on the role of c-di-AMP in the growth of *S. aureus* (11, 47), identification of amino acid transporters in *S. aureus* (9), the role of succinylome on MRSA sensitivity to beta-lactam antibiotics (17), and the role of pentose phosphate pathway and purine metabolism in MRSA beta-lactam resistance (12, 48) all reflect how these papers resonated with me and the way they have influenced my science throughout my career. Not only did these papers influence me, but they also influenced the field and have been followed up in several studies since their publication. My group is currently working on understanding and targeting the molecular mechanisms behind antimicrobial resistance in *Enterococcus faecium*, *Staphylococcus aureus*, *Klebsiella pneumoniae*, *Acinetobacter baumannii*, *Pseudomonas aeruginosa*, and *Enterobacter* spp., defined as the “ESKAPE pathogens” which are multi-drug resistant. I am looking forward to applying the tools, knowledge, principles, and scientific thinking I gained from these papers, as well as my previous mentors and collaborators, to my research in the coming years.
